# Bacterial biomechanics—From individual behaviors to biofilm and the gut flora

**DOI:** 10.1063/5.0026953

**Published:** 2020-10-28

**Authors:** Takuji Ishikawa, Toshihiro Omori, Kenji Kikuchi

**Affiliations:** 1 Department Finemechanics, Graduate School of Engineering, Tohoku University, Sendai 980-8579, Japan; 2 Department Biomedical Engineering, Graduate School of Biomedical Engineering, Tohoku University, Sendai 980-8579, Japan

## Abstract

Bacteria inhabit a variety of locations and play important roles in the environment and health. Our understanding of bacterial biomechanics has improved markedly in the last decade and has revealed that biomechanics play a significant role in microbial biology. The obtained knowledge has enabled investigation of complex phenomena, such as biofilm formation and the dynamics of the gut flora. A bottom-up strategy, i.e., from the cellular to the macroscale, facilitates understanding of macroscopic bacterial phenomena. In this Review, we first cover the biomechanics of individual bacteria in the bulk liquid and on surfaces as the base of complex phenomena. The collective behaviors of bacteria in simple environments are next introduced. We then introduce recent advances in biofilm biomechanics, in which adhesion force and the flow environment play crucial roles. We also review transport phenomena in the intestine and the dynamics of the gut flora, focusing on that in zebrafish. Finally, we provide an overview of the future prospects for the field.

## I. INTRODUCTION

Bacteria appeared on Earth more than 3 billion years ago. They inhabit a wide variety of locations and account for about 13% of the global biomass (Bar-On *et al.*, 2018). Ecologically, bacteria are at the bottom of the food chain and many protists rely on them. Bacteria also play crucial roles in the nitrogen and carbon cycles. In engineering, they are used for water purification in sewage treatment and for extracting metals from ores (biomining). Bacterial biofilms may cause deterioration in the performance of machinery, causing biofouling. The human gut harbors about 1 kg of bacteria, the number of which is about twofold that of human cells. The gut flora plays important roles in health and disease. Therefore, the study of bacteria is an important subject of scientific research.

**FIG. 1.**
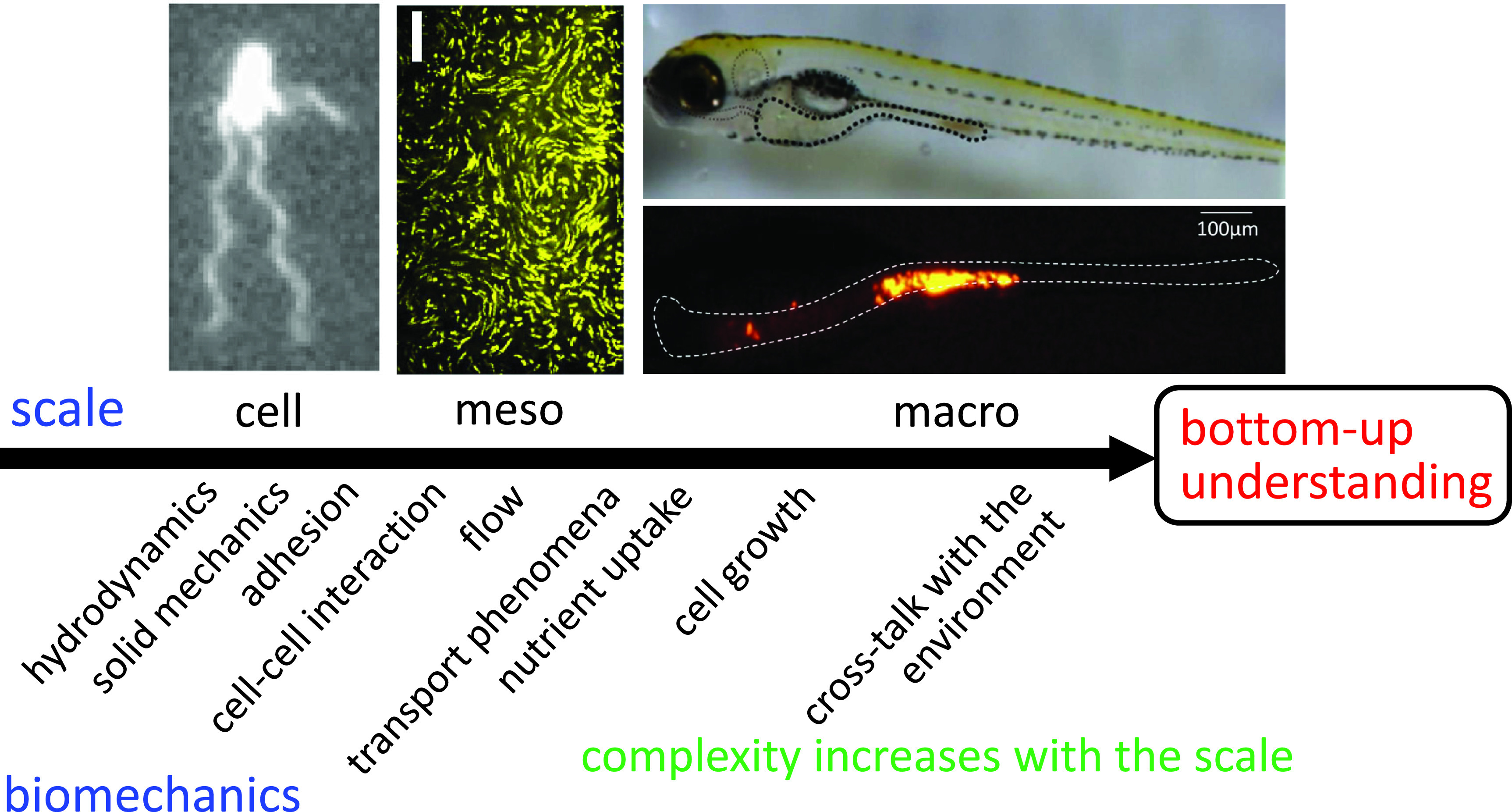
Schematic of a bottom-up understanding of bacterial biomechanics from the cellular to the macroscale. Top left, *Escherichia coli* cell; top middle, trajectories of tracers in a dense *E. coli* suspension; top right, zebrafish larva; and bottom right, fluorescent tracer particles in the intestine of zebrafish larva. The complexity of biomechanics increases with scale.

In the last decade, our understanding of bacterial biomechanics, such as adhesion on surfaces, collective swimming, clustering, and transport phenomena, has improved markedly. Former research revealed that biomechanics play a significant role in microbial biology. The knowledge obtained has been used to investigate more complex phenomena, such as biofilm formation and the dynamics of the gut flora. To understand such macroscopic phenomena, an understanding of the physical properties of the environment, such as nutrient concentration, diffusivity, and viscoelasticity, is needed. These macroscopic properties are governed by mesoscale microbial structures, which comprise intercellular interactions. Therefore, a bottom-up understanding of bacterial phenomena, i.e., from the cellular to the macroscale, is needed (Fig. 1).

In this Review, we focus on biomechanical aspects of bacterial phenomena, though microbiology has traditionally focused on the influence of chemical environments. Hydrodynamics and solid mechanics are used to discuss bacterial phenomena in all scales. Adhesion force is introduced in discussing the cell-surface interaction. Cell–cell interactions are important at the mesoscale because they determine the collective behaviors of cells. The transport of momentum, cells, and nutrients needs to be clarified at the mesoscale to extract macroscopic properties. At the macroscale, nutrient uptake, which governs the growth rate, must be discussed. Moreover, at the macroscale, cells' activities alter their environment and, in turn, their behaviors change. Thus, crosstalk between cells and their environment has to be discussed. Hence, the complexity in biomechanics necessarily increases as the scale increases (Fig. 1).

In Sec. II, we explain the behaviors of individual bacteria in the bulk liquid and on surfaces as the base of complex phenomena. The collective behaviors of bacteria in simplified environments are introduced in Sec. III. In Sec. IV, we discuss the biomechanics of biofilm formation, in which adhesion forces and the flow environment play crucial roles. In Sec. V, we discuss the gut flora in terms of biomechanics, focusing on that in zebrafish. In each section, we suggest other reviews as further reading. In Sec. VI, we discuss future prospects for this field.

## II. BIOMECHANICS OF AN INDIVIDUAL BACTERIUM

The individual behavior of a bacterium is the basis of their collective behaviors. We thus discuss the biomechanics of an individual bacterium in this section. In Sec. II A, we focus on a bacterium swimming in the bulk liquid. The physical environment of the intestine can be complex both rheologically and geometrically; therefore, the effects of solvent rheology and channel geometry on the swimming behaviors are discussed in Sec. II B. In Sec. II C, we focus on a bacterium adhered on a surface, because it is the beginning of biofilm formation. We suggest the following reviews as supplementary reading: Lauga (2016) reviewed bacterial hydrodynamics, Berg (2004) overviewed biological aspects of bacterial swimming, including chemotaxis, and Persat *et al.* (2015) explained the mechanical environment of surface-associated bacteria.

### A. Bacteria swimming in bulk liquid

Bacterial flagella, such as those of *Escherichia coli* (*E. coli*), are rotated by tiny molecular motors, and the estimated torque of the motors is 2000–5000 pN nm (Chen and Berg, 2000; Sowa *et al.*, 2003; Darnton *et al.*, 2007; Inoue *et al.*, 2008; Shimogonya, 2015). The torque generated by the motor is transmitted to a helical flagellar filament via a hook (Berg, 2003), and the rotation of helical flagella generates the propulsive force. As a result, *E. coli* (body length ∼2 *μ*m) swims at a velocity of about 20 *μ*m/s. Swimming motion can be broadly characterized by the Reynolds number, which indicates the ratio of inertia force to the viscous force. The Reynolds number of *E. coli* is on the order of 10^−5^, and so they live in the viscous dominant world.

Bacteria often have multiple flagella. The multiple flagella of *E. coli* form a single bundle when all flagella rotate in a counterclockwise direction, resulting in forward motion (Berg, 2004). When some flagella change the direction of rotation, the bundle unravels and the flagella separate. The biomechanics of flagellar bundling has been investigated (Kim *et al.*, 2003; Watari and Larson, 2010; Reigh *et al.*, 2012; Kanehl and Ishikawa, 2014). *E. coli* can adapt the frequency of alternation between tumbling and running, facilitating chemotaxis (Berg, 2004; Codling *et al.*, 2008; Goto and Nakai, 2016).

### B. Behaviors in complex environments

**FIG. 2.**
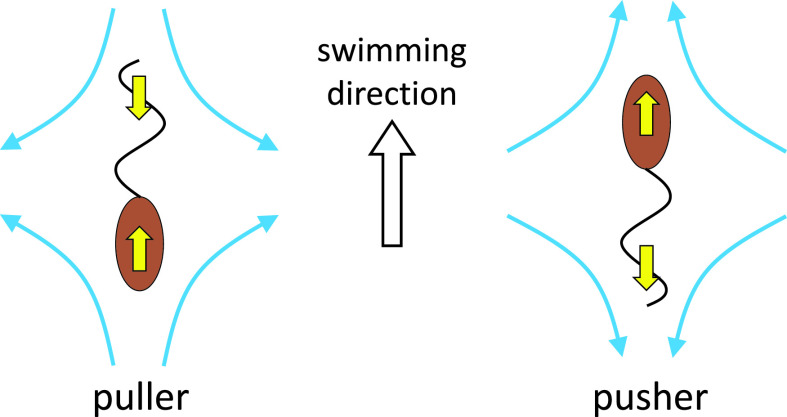
Schematics of a puller and a pusher. The puller has a flagellum in front of the cell body, while the pusher has a flagellum behind the cell body.

Bacteria can be attracted by a solid surface. Berke *et al.* (2008) showed that swimming bacteria tend to be attracted to surfaces, because the steady-state density increased 10-fold near surfaces. The attraction mechanism could be explained by the far-field hydrodynamic effect, which requires awareness of the difference between a pusher and a puller. The puller has a flagellum in front of the cell body, while the pusher has a flagellum behind the cell body. Figure 2 shows the flow fields far from the cell body; these are opposite between pullers and pushers and are not affected by cell shape. A pusher-type bacterium such as *E. coli* tends to orient parallel to and be attracted to surfaces due to the far-field hydrodynamic effect (Berke *et al.*, 2008; Lauga and Powers, 2009).

A pusher-type bacterium can be entrapped by a surface boundary due to the near-field alignment effect. In the vicinity of a surface boundary, *E. coli* draws a circular trajectory (Lauga *et al.*, 2006; Frymier *et al.*, 1995; Vigeant and Ford, 1997; Lauga *et al.*, 2006). Similar trajectories were also reported for the singly flagellated bacterium *Vibrio alginolyticus* (Goto *et al.*, 2005). The minimum separation distance between the cell and the surface has been estimated to be ten to several hundred nanometers (Frymier *et al.*, 1995; Vigeant and Ford, 1997; Vigeant *et al.*, 2002). The mechanism of entrapment by a surface has been explained by hydrodynamics and steric effects (Ramia *et al.*, 1993; Lauga *et al.*, 2006; Giacché *et al.*, 2010, Goto *et al.*, 2010; Giacché *et al.*, 2010). Notably, the circular trajectory found near a solid surface switches the direction near a free surface (Lemelle, 2010). When background shear flow is imposed, *E. coli* swimming near a wall drift perpendicular to the flow direction (Ishikawa *et al.*, 2014).

Some bacteria live in heterogeneous granular matter, such as soil, and the habitat is influenced by physical parameters including the granular size and shape (Young and Crawford, 2004; Or *et al.*, 2007). Brown *et al.* (2016) studied *E. coli* swimming in a monolayer of spheres on a surface. The circular orbits of *E. coli* on a flat wall were modified to long and straight runs, because the bacteria are unable to turn corners inside the crystal. Swimming in granular matter has also been analyzed using a simple microswimmer model (Volpe *et al.*, 2011; Chamolly *et al.*, 2017), in which straight swimming, random walking, entrapment in a closed orbit, or entrapment of cells were observed. These results illustrate the significant influence of geometric constraints on bacterial behaviors.

The effect of viscosity on bacterial swimming velocity has been investigated. The swimming velocity increases up to about twofold the viscosity of water but decreases monotonically with further increases in viscosity (Schneider and Doetsch, 1974; Greenberg and Canale-Parola, 1977). The decreasing tendency is caused by the constant torque of the molecular motor in the low-speed regime, and the additional viscous drag decreases the rotational velocity. The velocity increase may be related to the non-Newtonian property of fluid induced by the structure of polymer chains (Lauga, 2016).

### C. Bacteria adhered on surfaces

A single bacterium adhered on a wall can grow and divide and then become sessile and eventually construct multicellular structures, i.e., biofilms (Hall-Stoodley *et al.*, 2004). Thus, it is important to understand the biomechanics of bacterial adhesion (Persat *et al.* 2015).

Interactions between charged surfaces via a liquid medium can be understood based on the Derjaguin–Landau–Verwey–Overbeek (DLVO) theory of colloid stability (Verwey and Overbeek, 1948). Bacteria have acquired various adhesion strategies to overcome repulsive interactions. Many bacteria have pili, which are hair-like appendages distributed on the cell surface. Pili enhance bacterial adhesion by increasing the probability that a cell makes physical contact with a surface. Bacteria also secrete extracellular polymeric substances (EPSs). The EPS is composed of polysaccharides and other macromolecules and increases the affinity of bacteria to surfaces (Flemming and Wingender, 2010). Microscale surface patterns, such as submicrometer crevices (Friedlander *et al.*, 2013), nanoporous surfaces (Feng *et al.*, 2015), and bump lines (Yang *et al.*, 2019), have the potential to reduce the adhesion of bacteria.

The background flow also influences bacterial adhesion to surfaces. When the wall shear stress is sufficiently strong, the viscous drag acting on the cell body overwhelms the adhesion force and the cell is washed away. Some studies have reported that bacteria can establish a catch bond, in which the dissociation lifetime increases with the tensile force applied to the bond, i.e., the adhesion force increases with the background flow (Thomas, 2008). Nilsson *et al.* (2006) found that type I fimbriae attachment of *E*. *coli* has a shear threshold for binding, below which bacteria do not adhere. This tendency benefits not only biofilm formation but also the gut flora, because bacteria can resist the peristaltic motion of the intestine.

## III. COLLECTIVE BEHAVIORS OF BACTERIA

In this section, we explain the collective behaviors of bacteria in simplified settings, to promote understanding of bacterial behaviors in biofilms and the gut. In Sec. III A, collective swimming and coherent structures of bacteria in dense suspensions are introduced. Transport phenomena, such as mass transport and viscosity, are important for macroscopic cell behaviors and growth and are discussed in Sec. III B. In Sec. III C, we discuss colony pattern formation on agar gels, which is important for understanding biofilms. We suggest the following as supplementary reading: Elgeti *et al.* (2015) reviewed collective behaviors of microswimmers from the physical perspective and Jacob *et al.* (2004) focused on self-organization of bacterial colonies. In this Review, bioconvection of a bacterial suspension is not discussed. Bioconvection is the biological analogue to a thermal convection and results in geometric patterns. See Hill and Pedley (2005) and Pedley and Kessler (1992).

### A. Collective swimming

In dense bacterial suspensions, such as 10^10^ cell/ml, bacteria tend to swim in a similar direction to their neighbors and generate mesoscale coherent structures (Dombrowski *et al.*, 2004; Ishikawa *et al.*, 2011; Dunkel *et al.*, 2013). The mechanism of this collective motion has been investigated using continuum models, which do not consider near-field cell–cell interactions. By considering the alignment of cells in the stretching direction of background flow, the continuum models reproduced turbulent bacterial spatiotemporal motion (Ramaswamy, 2010; Koch and Subramanian, 2011; Marchetti *et al.*, 2013; Wensink *et al.*, 2012). Therefore, coherent structures can be reproduced by far-field fluid interactions, and near-field cell–cell interactions may not be important.

Discrete models have been employed to investigate the collective motion of individual cells. The hydrodynamic interactions among multiple cells in near- and far-fields have been elucidated (Ishikawa and Pedley, 2008; Ishikawa *et al.*, 2008; Evans *et al.*, 2011; Lambert *et al.*, 2013; Potomkin *et al.*, 2013; Zottl and Stark, 2014). The discrete models also successfully reproduced the turbulent bacterial spatiotemporal motion; however, questions remain regarding the role of near-field fluid mechanics in collective motion.

**FIG. 3.**
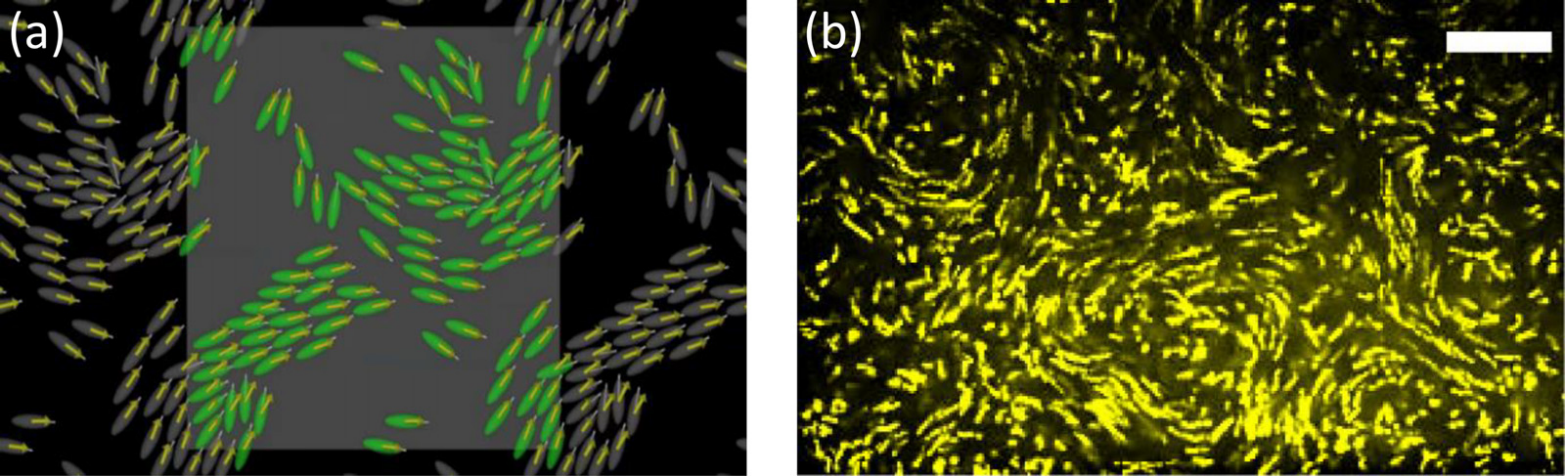
Collective motion of bacteria in a dense suspension. (a) Numerical simulation of collective swimming of prolate squirmers in a monolayer suspension. Yellow arrows, velocity vectors; and gray lines, orientations. The areal fraction of cells is 0.3, and the cells are pushers (*β* = −0.3). (Problem settings are identical to Kyoya *et al.*, 2015.). (b) Trajectories of tracer particles in a dense *E. coli* suspension. Scale bar, 50 *μ*m. (Settings are identical to Ishikawa *et al.*, 2011.)

Kyoya *et al.* (2015) solved the Stokes flow around hydrodynamically interacting prolate squirmers in a monolayer suspension. Weak pushers, such as *E. coli*, generated coherent structures [Fig. 3(a)]. To separate the effects of near- and far-field hydrodynamic interactions, they performed physically inconsistent but sophisticated trial simulations. The flow field of Stokes flow can be expressed by a boundary integral equation, in which the integral of traction forces over all squirmer surfaces appears. Instead of performing it rigorously, they performed the integral for cells only in the near field, although it was not physically consistent. The flow generated by far-field cells disappeared, and the resultant structure was generated solely by near-field cell–cell interactions. Kyoya *et al.* showed that the coherent structures found in bacterial baths are generated mainly by near-field cell–cell interactions.

### B. Transport phenomena in bacterial suspensions

We discuss here the transport of momentum, cells, and substances in a bacterial suspension (Ishikawa, 2009). The transport of momentum is closely related to the rheological properties of the suspension and so affects the flow field. The transport of cells governs their distribution in the suspension. Transport of substances, such as oxygen and nutrients, determines the growth rate of cells. Thus, transport phenomena are important for understanding short- and long-term bacterial behaviors.

**FIG. 4.**
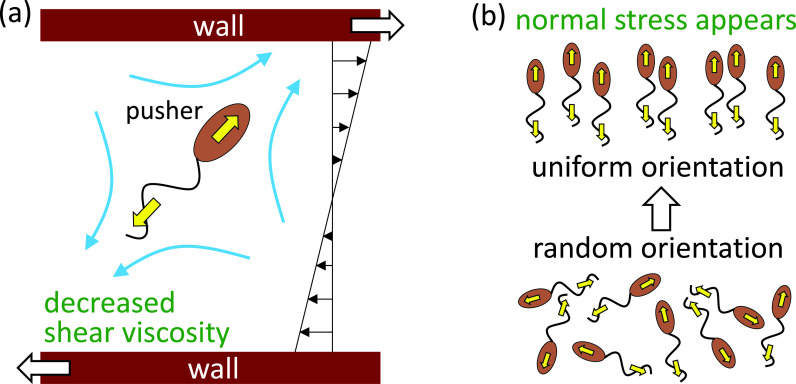
Schematics of the stress field generated by pushers. (a) A pusher with the given orientation helps the wall movement, and thus the shear viscosity is decreased. (b) Normal stress appears when orientation of pushers is anisotropic.

The bulk stress tensor in a Newtonian fluid can be given by Newton's law of viscosity. When small particles are suspended in a Newtonian fluid, however, an additional contribution to the bulk stress tensor is generated. The additional stress is termed a particle stress tensor and can be calculated from the traction forces and velocities on the particle surface (Batchelor, 1970). The particle stress tensor governs the rheological properties of the suspension, such as its viscosity and normal stress differences. Ishikawa and Pedley (2007) investigated the shear viscosity in a semi-dilute suspension of active cells. They showed that, when the active cells are aligned in a certain direction, the shear viscosity is altered from that of non-motile cells. For example, when a pusher is placed relative to the shear flow as shown in Fig. 4(a), it helps the wall movement and the shear viscosity is decreased. The change in the shear viscosity is opposite between the pullers and pushers (cf. Fig. 2). Also, when the cells are aligned, normal stress differences appeared in the suspension, as shown in Fig. 4(b). If forming or destroying the cells' alignment requires some time, the stress tensor relaxes with time (Ishikawa *et al.*, 2007). Thus, bacterial suspensions have non-Newtonian rheological properties.

The shear viscosity in a bacterial suspension can be decreased below the viscosity of solvent fluid (Hatwalne *et al.*, 2004; Haines *et al.*, 2009; Ryan *et al.*, 2011). Giomi *et al.* (2010) predicted a superfluid phase with vanishing viscosity for strongly active swimmers. López *et al.* (2015) showed, using *E. coli*, that a bacterial suspension displays a superfluid-like transition at which viscous resistance to shear vanishes. Therefore, the activity of pusher bacteria organized by shear can overcome viscous dissipation.

Macroscopic transport of cells can be modeled by a continuum model that incorporates advection, diffusion, and growth effects (Pedley and Kessler, 1992). The advection effect can be expressed by the drift velocity, which is a function of background flow, surface boundaries, and chemotaxis (Berg, 2004; Ishikawa, 2012; Lauga, 2016). The diffusion effect may be expressed using a diffusion tensor, which can be caused by the intrinsic randomness of biological processes and cell–cell hydrodynamic interactions (Cates, 2012). Ishikawa and Pedley (2007) investigated the hydrodynamic effect in a semi-dilute regime. The translational diffusivity was inversely proportional to the cell-number density, while the rotational diffusivity was proportional to the number density.

In a dense bacterial suspension, transport of tracer particles is significantly increased. Wu and Libchaber (2000) showed, using *E. coli*, that the diffusivity of 10-*μ*m-diameter tracer particles is three orders of magnitude larger than Brownian diffusivity. This marked increase in diffusivity is caused by the turbulent-like flow structure [Fig. 3(b)]. A similar increase in tracer diffusivity has been reported by others (Sokolov *et al.*, 2009; Ishikawa *et al.*, 2011; Jepson *et al.*, 2013).

### C. Colony pattern formation

The study of bacterial colonies is important for understanding biofilms (Jacob *et al.*, 2004). Bacteria show adaptable collective responses and form colonies with geometric patterns. Colony dynamics comprise the trajectories of aggregates that grow, move, and reproduce simultaneously. Wakita *et al.* (1997) classified the colony patterns of *Bacillus subtilis* into five types: (i) diffusion-limited aggregation-like, (ii) Eden-like, (iii) concentric ring-like, (iv) disk-like, and (v) dense branching morphology-like. The morphology was dependent on the substrate softness and nutrient concentration. Therefore, transport phenomena in bacterial colonies strongly affect their morphology.

Bacteria in colonies can migrate collectively on surfaces and generate large swirls and jets. Such swarming involves several cellular processes, such as changes in the levels of key proteins, intercellular chemical communication, and mechanical factors (Beer and Ariel, 2019). Therefore, swarming can be distinguished from collective swimming (Sec. III A), in which cellular processes do not play a major role. Ariel *et al.* (2015) investigated the motion of bacteria within a swarm and found that bacterial spreading was not diffusive but superdiffusive.

To describe colony dynamics, several mathematical models have been proposed. Mimura *et al.* (2000) proposed a model by combining a reaction diffusion system of nutrient dynamics, the nucleation theory of aggregate generation, and individual-based dynamics of the motion and growth of aggregates. The model consistently reproduced the experimentally observed branching patterns and effects of the initial nutrient concentration. The model was enhanced to take into account active and inactive bacterial cells (Matsushita *et al.*, 2004). Gerlee and Anderson (2007) proposed a hybrid cellular automaton model of colony growth, in which colony growth is limited by the concentration of a nutrient, which inhibits cell division if it falls below a threshold. Dong and Klumpp (2018) used a cellular automaton model to investigate colony pattern formation under the differential adhesion hypothesis and in the presence of cell proliferation.

Farrell *et al.* (2013) assumed that bacteria interact purely mechanically, by pushing each other away as they grow and consume a diffusing nutrient. They showed that mechanical interactions can explain the transition between circular and branching colonies. The importance of mechanics is also emphasized by the study of Ghosh *et al.* (2015), in which repulsive forces generated phase-separated patterns in the growing colony. Although these mathematical models clarified aspects of colony growth, our understanding remains limited. For example, the effect of swarming on transport phenomena in the colony is unclear. Future studies are expected to provide a more quantitative description of colony growth.

## IV. BIOFILM FORMATION

Biofilms are surface-associated microbial communities encased in a self-secreted matrix of extracellular polymeric substances (EPSs) (Jang *et al.*, 2017). Bacteria typically live in biofilms as their natural habitat (Drescher *et al.*, 2013), and biofilms account for a large proportion of bacterial biomass. For example, biofilms are the sources of infections associated with catheters and implanted devices (Brooks and Flint, 2008). In industry, biofouling and biocorrosion by biofilms are responsible for pipe-clogging and product contamination (Campoccia *et al.*, 2013; Jang *et al.*, 2017). Biofilms are different from planktonic bacteria, exhibiting markedly greater resistance to antibiotics and external stressors (Nguyen *et al.*, 2011).

Both the chemical and biomechanical environments play significant roles in biofilm formation, as explained in Sec. IV A. The surrounding hydrodynamics can generate flow-shaped biofilms, called streamers (Rusconi *et al.*, 2010) (Sec. IV B). The deformation of biofilms depends on their physical properties, which are reviewed in Sec. IV C. To understand the physical mechanism of biofilm formation several mathematical models have been developed. We review them in Sec. IV D. We suggest the following as supplementary reading: Dufrêne and Persat (2020) reviewed bacterial mechanobiology, Persat *et al.* (2015) focused on mechanical aspects of biofilm formation, and Even *et al.* (2017) reviewed the mechanical properties of biofilms.

### A. Biofilm formation

Biofilm formation is influenced by many factors, such as species, genotype, pH, nutrient, water content, and the presence of other microbial species (Albareda *et al.*, 2006). Bacteria within biofilms can communicate to control their growth rate, i.e., quorum sensing. Shrout *et al.* (2006) reported that quorum sensing in *P. aeruginosa* exerted nutritionally conditional control of biofilm development by regulating swarming motility. Also, distant bacterial biofilms can coordinate their growth dynamics to resolve competition for limited resources (Liu *et al.*, 2017). Biofilm structures can thus result from the interaction between the environment and individual cells.

Hydrodynamics also influence biofilm formation, because they govern drag force and mass transport (Liu and Tay, 2002). Strong shear stress may lead to breakage of a biofilm, which induces dispersion of the biofilm to new territories. Stoodley *et al.* (1999) observed biofilm growth of gram-negative species under turbulent and laminar flows and found patchy biofilms in laminar flow and patches of ripples and elongated streamers in turbulent flow. Simoes *et al.* (2007) investigated biofilm formation by *Pseudomonas* under turbulent and laminar flows and found that turbulent flow-generated biofilms were metabolically more active and had higher cell densities than laminar flow-generated biofilms. Vieira *et al.* (1993) measured the mass transfer coefficients in *Pseudomonas* biofilms at different fluid velocities; the mass transfer rates decreased more quickly as the fluid velocities increased, and lower internal diffusivities resulted in lower final thicknesses of the biofilms.

Pathogens also take advantage of biofilm formation. For example, plant-associated bacteria can sense plant exudates released from specific sites and aggregate via chemotaxis. Yao and Allen (2006) showed that directed motility, not simply random motion, is required for full virulence and that chemotaxis is an important trait for virulence and pathogenic fitness in plant pathogens. Fujishige *et al.* (2006) used flagella-less *Sinorhizobium* mutants and found that nodule formation was significantly delayed without cell motility. These results show that cell motility plays important roles in the initiation, maturation, and spread of biofilms and colonization of new habitats. Moreover, the surrounding flow field influences biofilm formation by pathogens. Kostenko *et al.* (2010) investigated the biofilm morphology and tolerance of *Staphylococcus* to antibiotics under oscillating shear stress as a model of bacterial infections in the blood system. They found that pulsatile flow promotes the formation of more tolerant biofilms and engenders difficult-to-treat infection sites.

### B. Streamers—biofilm formation under flow

In the last decade, the link between hydrodynamics and complex biofilm structures has been investigated experimentally and theoretically. A remarkable example is filamentous flow-shaped biofilms, i.e., streamers (Rusconi *et al.*, 2010). Streamers are frequently observed in soil-like porous environments and industrial filters. Biofilm streamers may cause rapid clogging in porous materials (Drescher *et al.*, 2013). Flow-induced shedding of the extracellular matrix from surface-attached biofilms generates a sieve-like network that exponentially accelerates clogging.

The impact of fluid flow has been studied using microfluidic devices (Biswas *et al.*, 2016). Rusconi *et al.* (2010) investigated streamer formation in curved microchannels under laminar flow. The streamers were spatially localized to the middle plane of the curved channel and connected only at the inner curve during each turn, where a secondary vortical flow was observed. The effect of secondary flow on biofilm formation was evaluated by Marty *et al.* (2014). They discussed the role of secondary flow in streamer formation in three steps. The first step was shear-enhanced adhesion following the streamlines toward the surfaces at the pore entrance. The second step was formation of filaments. EPSs form filaments and, as the third step, the filaments form a net, which captures floating bacteria. Hydrodynamics profoundly affects how bacteria compete and evolve in biofilms (Coyte *et al.*, 2017). Thus, hydrodynamical modeling of biofilm formation is important for predicting and controlling their development and detachment.

### C. Physical properties of biofilms

Even *et al.* (2017) reviewed the mechanical properties of biofilms, which affect the deformation and breakage of biofilms. Several measurement techniques, such as rheometry, uniaxial compression, atomic force microscopy, and hydrodynamic load, have been used to assay the macroscopic mechanical properties of biofilms. These techniques revealed that biofilms are viscoelastic. However, the elastic modulus and viscosity values of the biofilm strongly depend on the experimental settings. The effective shear modulus ranges widely from 10^−2^ to 10^6^ Pa (Even *et al.*, 2017). The storage and loss moduli *G′* and *G″* were also measured (Lieleg *et al.*, 2011). The results suggested that biofilms behave as viscoelastic solids rather than viscoelastic liquids, because *G′* was about tenfold larger than *G″*. However, this was dependent on the time scale of the experiment (Galy *et al.*, 2012). Strain-hardening and viscoplastic effects have also been reported (Hollenbeck *et al.*, 2016).

The diffusivity inside biofilms is important because it modulates mass transport. Stewart (2003) discussed the influence of diffusion on the chemistry and biology of biofilms. He measured the effective diffusion coefficient in the biofilm *D_e_* and showed that *D_e_* is reduced compared to that in water (*D_aq_*) because of the presence of microbial cells and extracellular polymers. The reduction ratio *D_e_*/*D_aq_* was estimated to be 0.6 for light gases (e.g., oxygen, nitrous oxide, carbon dioxide, or methane) and 0.25 for most organic solutes. For light gases, we have the scaling *D_e_ *∼ *L*^2^/*T* ∼ 0.6*D_aq_*, where *L* is the length scale, *T* is the time scale, and *D_aq_* ∼ 10^−9^ m^2^/s. The timescale for diffusive equilibration can be scaled as *T* ∼ *L*^2^/(0.6*D_aq_*), which ranges from one second to tens of minutes when the length scale is tens to thousands of micrometers. Also, topological heterogeneity does not alter the fundamental phenomena of diffusive transport in a biofilm.

Physical properties of biofilms composed of multi-species communities have been investigated. A dual-species biofilm, composed of *Vibrio* and *E. coli*, showed competition at the interface between the two species (Abriat *et al.*, 2020). The viscoelastic properties of the dual-species biofilm were dominated by *Vibrio*, because *Vibrio* could form a mature biofilm faster than *E. coli*. A dual-species biofilm of *Bacillus* and *Pseudomonas* was investigated by Abriat *et al.* (2019). Mechanical properties of the biofilm were dominated by *Pseudomonas*, and the study of the planktonic and biofilm growths for each species revealed that *Pseudomonas* grew faster than *Bacillus*. These studies illustrate the importance of growth kinetics in the bacteria competition for the interface in a dual-species biofilm. Mixed-species cultures grow biofilms that are more robust and protective to bacteria than biofilms grown from single-species cultures (Burmolle *et al.*, 2006; Wilking *et al.*, 2011; Gordon *et al.*, 2017). Paramonova *et al.* (2009) investigated the influence of hydrodynamics on oral biofilms composed of single- and multi-species, because mechanical removal of oral biofilms is accepted as the best way to prevent caries and periodontal diseases. They found that multi-species biofilms were stronger than single-species biofilms. In response to increased hydrodynamic shear, biofilm strength decreased and architecture changed from uniform carpet-like to fluffy with a higher thickness.

### D. Modeling of biofilm formation

To simulate biofilm formation, several mechanical models have been developed. There are two major types of biofilm models; individual particle-based and continuum multiphase flow models. These are described in the following.

A particle-based biofilm model is focused on individual cell behaviors in an extracellular matrix. Cells are typically represented by Lagrangian spheres in an Eulerian liquid domain. Biomass accumulation and growth are simulated as new cells are added to the biofilm. Picioreanu *et al.* (2004) developed a particle-based biofilm model coupled to a reaction-diffusion equation. The growth of biomass was given by the reaction rate, which was described by the Monod equation. In order to express the boundary condition of mass flux through the biofilm surface, a concentration boundary layer can be introduced (Xavier *et al.*, 2005). Dillon *et al.* (1996) developed a fluid-structure interaction model by the immersed boundary method. Cell–cell and cell-wall adhesions were modeled by elastic springs between points on each adherent entity. The total force acting on a particle is then transmitted to the fluid using an immersed boundary method.

**FIG. 5.**
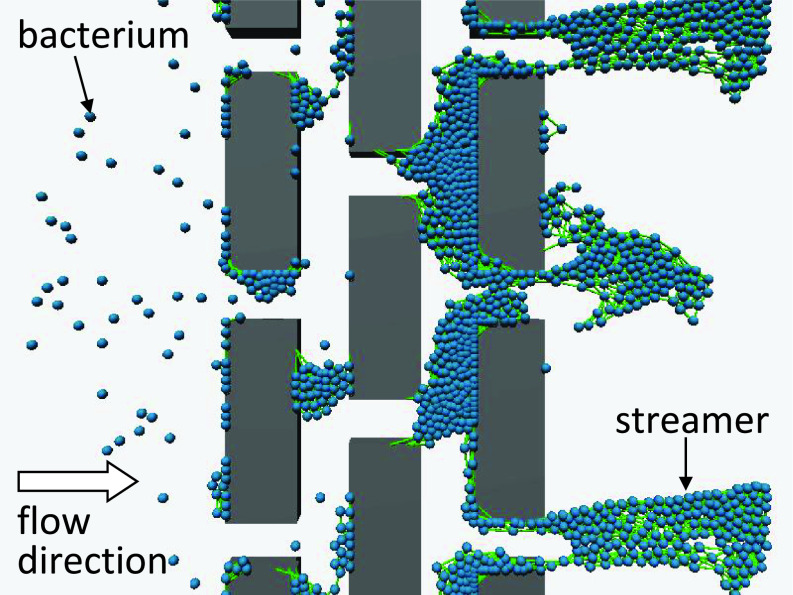
Particle-based simulation of the biofilm. A bacterial suspension flows through blocks, and streamers are formed on the downstream side. Spheres represent individual bacteria that have adhered to each other and the blocks.

These particle-based models reproduced three-dimensional two-species biofilm formation and predicted the spatiotemporal distributions of bacteria and substrates (Picioreanu *et al.*, 2004; Xavier *et al.*, 2005). For example, Xavier *et al.* (2005) simulated the formation of biofilms by polyhydroxybutyrate (PHB)- and EPSs-producing organisms. The PHB-producing organisms achieved dominance by supplying substrate intermittently in feast/famine cycles, as observed experimentally. Kitamura *et al.* (2019) reproduced streamers formed in a microchannel with blocks (Fig. 5). Particle-based modeling is a bottom-up approach and can be extended to multispecies biofilms.

Biofilms can also be simulated by continuum multiphase flow modeling (Albero *et al.*, 2014; Batchelor *et al.*, 2018; Cogan and Keener, 2004; Horn and Lackner, 2014; Picioreanu *et al.*, 2004; Rittmann, 1982; Taylor and Jaffe, 1990; Winstanley *et al.*, 2011; Zhang *et al.*, 2008). Bacterial biofilms may be modeled as a polymer solution immersed in a Newtonian liquid. Biofilm motion and growth are described by mass conservation of the polymer phase, solvent phase, and nutrients, as well as by momentum conservation in both the polymer and solvent phases. Time-dependent growth of biofilms is then simulated by coupling these equations. The models provide insight into biofilm development, such as biofilm dynamics in shear flow (Zhang *et al.*, 2008) and the relationship between osmotic pressure and biofilm morphology (Cogan and Keener, 2004).

## V. GUT FLORA

The number of bacteria in the intestine can be much larger than the total number of human cells (Guarner and Malagelada, 2003). Gut bacteria form a complex ecosystem, known as the gut flora or microbiota, and play important roles in homeostasis and health. The human gut flora has metabolic activity, tropic and immunologic effects, and prevents colonization by pathogens. The microbiota is also linked to disorders such as obesity, colon cancer, inflammatory bowel disease (IBD), and cardiovascular diseases (CVDs). Therefore, predicting and controlling the microbiota are important in medicine and biology.

Biomechanics can be a strong tool for elucidation of gut flora composition and activity, because the distribution and growth of bacteria are dependent on transport phenomena. In this section, we introduce the role of the gut flora in health (Sec. V A). Heterogeneous microbial distributions and the effect of hydrodynamics are discussed in Sec. V B. Zebrafish models of the gut flora have been developed, and so we describe the gut flora of zebrafish (Sec. V C). In Sec. V D, we cover mathematical modeling of the gut flora. We suggest the following as supplementary reading: Guarner and Malagelada (2003) reviewed the gut flora in health and disease, Donaldson *et al.* (2016) explained the spatial distribution of gut microbiota, Chahill (1990) focused on the bacterial flora of fish, and Song *et al.* (2014) described modeling of microbial community dynamics.

### A. Role of the gut flora

Approximately 10^13^–10^14^ bacterial cells and 15 000–36 000 bacterial species reside in the gut lumen (Sartor, 2008). Greater than 99% of the gut flora is composed of four major bacterial divisions—Firmicutes, Bacteroidetes, Proteobacteria, and Actinobacteria (Frank *et al.*, 2007). Enterobacteriaceae, such as *E. coli*, are relatively minor components of the division Proteobacteria (8% of all gut bacteria).

The microbiota produces several nutrients, including short-chain fatty acids (SCFAs), B vitamins, and vitamin K. SCFAs are fermented and produced from dietary fiber by the gut microbiota, and they modulate host health by influencing gut barrier function, glucose homeostasis, immunity, appetite, and obesity (Chambers *et al.*, 2018). Lactic acid bacteria (LAB) synthesize vitamin K and water-soluble B vitamins. Many herbivores are dependent on the digestion of plant material (e.g., cellulose, hemicellulose, and pectin) by the gut microbiota (Hanning and Diaz-Sanchez, 2015). Thus, the gut microbiota contributes to the production of dietary components.

Symbiosis with gut flora has both positive and negative effects on host health. The gut flora is linked to pathological disorders, such as allergies, obesity, colon cancer, IBD, and CVD (Zhnag *et al.*, 2015). Allergic disease in infants is caused by delayed establishment of *Bifidobacterium* and *Lactobacillus* in the gut, which regulate allergic-type immune responses (Kalliomäki and Isolauri, 2003). Fewer *Bacteroidetes* and more *Firmicutes* in the gut microbiota facilitate the extraction of calories from ingested dietary substances and promote their storage in adipose tissue, leading to obesity (DiBaise, *et al.*, 2008). A decrease in gut microbial diversity caused by a shift in the balance between commensal and potentially pathogenic microorganisms is associated with inflammatory bowel diseases, including Crohn's disease and ulcerative colitis (Ni *et al.*, 2017). SCFAs, which are produced by the gut microbiota, are also linked to the risk of CVD. Systolic and diastolic blood pressures are associated with decreased butyrate and plasminogen activator inhibitor-1 levels in early pregnancy (Chambers *et al.*, 2018).

The role of gut microbial biofilms in colorectal cancer is reviewed by Hold and Allen-Vercoe (2019). Initially, small aggregates of bacterial cells attach to mucosal surfaces. Then, mature biofilms develop when these adherent microcolonies become encapsulated in a self-secreted polysaccharide matrix. Dejea *et al.* (2014) identified invasive polymicrobial bacterial biofilms nearly universally on right-sided tumors but on only 12% of left-sided tumors. The results illustrate that the mucosa-associated microbial community is an important factor in colorectal cancer pathogenesis, particularly in the proximal colon. In addition, a number of healthy individuals also harbored mucosal biofilms. The complex microbial communities of the gut microbiota reside over the intestinal mucus as exopolysaccharide-coated biofilms, which disperse planktonic bacteria (Buret *et al.*, 2019). The mucosal biofilm bacteria differ phylogenetically and metabolically from those living in a planktonic state (Beatty *et al.*, 2017; Macfarlane *et al.*, 2005).

The formation of biofilms also protects the bacterial population from host immune responses and antibiotics by secretion of EPSs and binding bacteria together in layers (Swidsinski *et al.*, 2005). It can prevent the loss of useful secretions and nutrients from the population. IBD is a group of intestinal disorders in which prolonged inflammation occurs in the digestive tract. Biofilm formation in the digestive tract has an adverse effect on the immune response of the host. There is no satisfactory and safe treatment option for IBD. Therefore, the current research aims to disrupt biofilms in IBD and concentrates on improving the drug (Srivastava *et al.*, 2017).

One clear limitation in targeting the distribution of intestinal flora as the cause of chronic disease is the possibility that the inciting microbiota is no longer active at the time the disease is identified, perhaps because of a gradual shift or change in the intestinal environment. Therefore, real-time evaluation of gut motility and microbial diversity would be likely to clarify the causes of intestinal disorders.

### B. Dynamics of the gut flora

Studies of microbiota spatial organization in the vertebrate gut have revealed functional relationships between biogeography and health, driven by coincident revolutions in imaging and sequencing technologies (Tropini *et al.*, 2017). It is becoming clear that spatial redistribution of the microbiota can be a common and functionally relevant feature of chronic inflammatory diseases. The density and composition of the microbiota are affected by chemical, nutritional, and immunological fields along the gut (Thursby and Juge, 2017). In the small intestine, there are typically high levels of acids, oxygen, and antimicrobials and a short transit time. These properties limit bacterial growth such that only rapidly growing anaerobes with the ability to adhere to epithelia mucus would survive (Donaldson *et al.*, 2015). In contrast, colonic conditions support a dense and diverse community of bacteria, mainly anaerobes, where *Prevotellaceae*, *Lachnospiraceae,* and *Rikenellaceae* are dominant. Spatial metagenomics can be a strong tool to study microbial biogeography in complex habitats. Sheth *et al.* (2019) characterized the spatial organization of a microbiome by spatial metagenomics and revealed heterogeneous microbial distributions. They showed that phylogenetically clustered local regions of bacteria were associated with a dietary perturbation.

Heterogeneous microbial distributions have been found in various vertebrate guts. Bacteria within the mouse gut migrate to closer proximity to the epithelium when diet lacks microbiota accessible carbohydrates (Earle *et al.*, 2015). Suzuki and Nachman (2016) investigated microbial communities from ten different segments of the gastrointestinal tract (mouth, esophagus, stomach, duodenum, ileum, proximal cecum, distal cecum, colon, rectum, and feces) in wild house mice. The lower gastrointestinal tract had a greater relative abundance of anaerobic bacteria and greater microbial diversity relative to the upper gastrointestinal tract. A large-scale gene sequencing was performed for newborn piglets. Using intestinal digesta, Liu *et al.* (2019) showed that the six intestinal segments could be divided into three parts; in the duodenum-jejunum section, the most abundant genera included *Lactobacillus* and *Bacteroides*; in the ileum, *Fusobacterium* and *Escherichia*; and in the cecum-rectum section, *Prevotella*. Schlomann *et al.* (2018) observed bacterial distribution patterns throughout the intestinal volume of live larval zebrafish. Fluorescently tagged strains of seven bacterial symbionts showed large differences in both cohesion and spatial distribution. Significant spatial heterogeneity in composition, diversity, and species of gut microbiota was also found in wild folivorous flying squirrels (Lu *et al.*, 2014).

Hydrodynamic forces in the colon, along with colonic water absorption that manifests as transit time, exert a significant impact on microbiota density and composition. Arnoldini *et al.* (2018) explained the hydrodynamic effect on colonic pH, which directly affects microbiota competition for food. They also discussed the mixing dynamics of luminal content by wall contractions and its implications for bacterial growth and density. Active mixing of colonic contents was found to be crucial to prevent washout of bacteria. Transport phenomena of bacteria across and along the gastrointestinal tract were experimentally investigated by Takahashi and Sakaguchi (2006). They injected fluorescently labeled viable bacteria into the proximal colon of the guinea pig to observe transport in the large intestinal lumen. Their results showed that bacteria were transported along the radial and longitudinal axes of the intestine and even transported back to the cecum. They speculated that the spreading of bacteria upstream may be caused by the backward flow of dietary residue in the furrow of the proximal colon (Takahashi and Sakaguchi, 2000).

The effect of viscosity on the growth of gut microbiota at physiological conditions was investigated by Tamargo *et al.* (2018). They performed *in vitro* experiments using a gastrointestinal simulator, in which the viscosity was controlled by the agar concentrations. The results indicated that changes in intestinal viscosity selectively modify the microbiota composition. Cremer *et al.* (2016) developed a fluidic channel that allows mimicking active contractions of the colonic wall. They found that repeated contraction was crucial to prevent bacterial washout and maintain a steady-state bacterial population, which illustrates that flow and mixing play a major role in shaping the microbiota of the colon.

Parthasarathy (2018) visualized swimming behaviors of bacteria in the larval zebrafish gut by using a light sheet microscope. Wiles *et al.* (2020) also investigated the spatial organization and dynamics of bacterial populations within the larval zebrafish gut. They found that a proinflammatory *Vibrio* symbiont governs its own spatial organization using swimming motility and chemotaxis. The motility did not enhance its growth rate but rather promoted its persistence by resisting the intestinal flow. In contrast, nonmotile *Vibrio* mutants surrendered to the intestinal flow. Consequently, nonmotile and nonchemotactic mutants were susceptible to intestinal expulsion.

### C. Zebrafish as a model of the gut flora

The use of mammalian *in vivo* models of gastrointestinal chyme transport is limited by the physical space needed for housing and by the labor-intensive surgery and invasive imaging techniques used (Field *et al.*, 2009). As a model organism, the zebrafish, *Danio rerio*, has been widely used to investigate the roles of environmental, genetic, or chemical perturbations in gastrointestinal motility and transport. Zebrafish genetics, organ physiology, and metabolism are similar to those of human (Barbazuk *et al.*, 2000; Quinlivan and Farber, 2017; Zang *et al.*, 2017). The > 500 mutant phenotypes—which are associated with hematopoietic, cardiovascular, kidney, and other-organ disorders—of zebrafish enable development and genetics studies (Dooley and Zon, 2000). The zebrafish also has high fertility and a short generation time (Freifeld *et al.*, 2017).

Real-time *in vivo* observation enables analysis of the developmental morphology and function of organs. The transparent larval body facilitates noninvasive real-time observation of organ inner structures. *In vivo* digital motion analysis in *Danio rerio* zebrafish (3–7 days post fertilization [dpf]) showed that the peristaltic frequency was in the range of 0.015–0.025 Hz, while the velocity of peristaltic wave propagation was around 15 *μ*m/s (Holmberg *et al.*, 2003). The viscosity of fluid in the anterior intestine of zebrafish larvae was measured using oscillating magnetic prolate fluorescent particles as 0.5 mPa s, i.e., approximately fivefold larger than that of water (Taormina *et al.*, 2017). By feeding fluorescent polystyrene microspheres mixed with powdered feed to a zebrafish larva at 7 dpf, intestinal transit was observed under a fluorescent microscope (Field *et al.*, 2009). Even 12 h after feeding, 72% of microspheres remained, while they were absent 24 h after feeding. Cosshiaro *et al.* (2013) showed that 50% of zebrafish larva at 7 dpf eliminated the injected material in 12 hours.

**FIG. 6.**
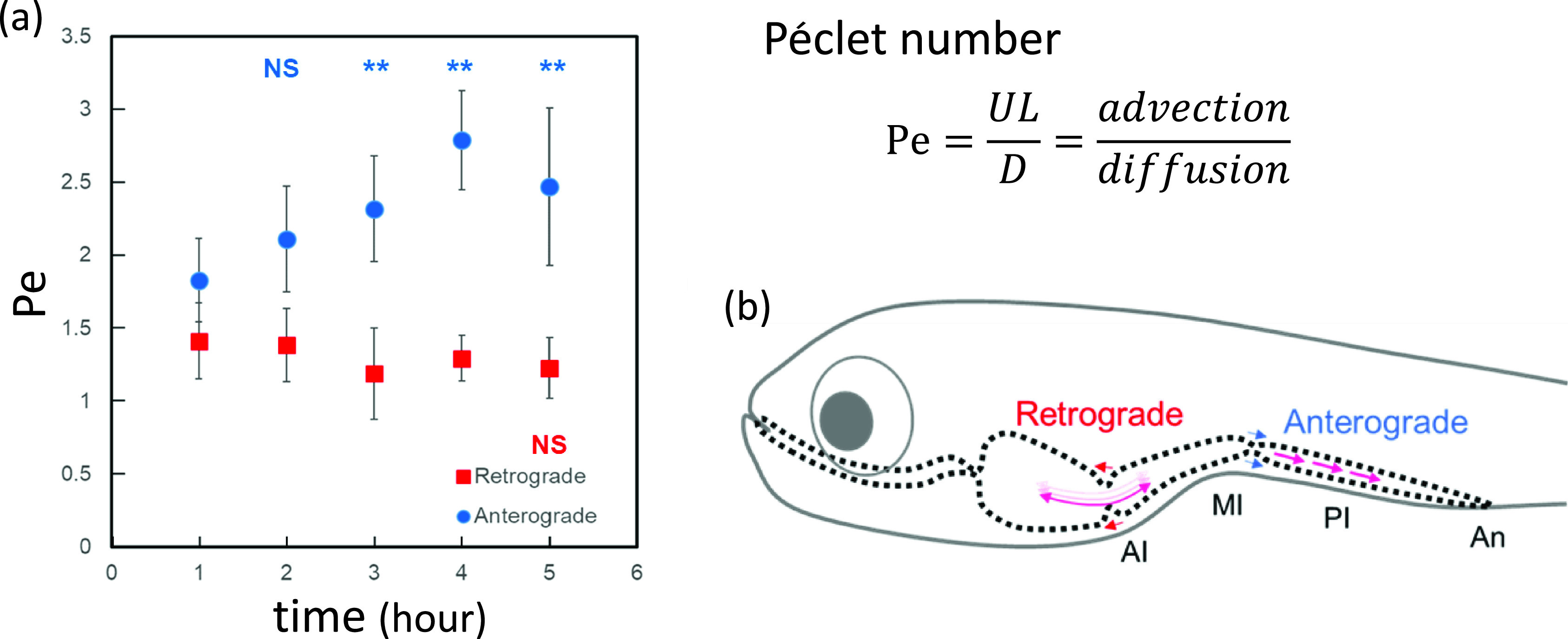
Mechanical roles of anterograde and retrograde intestinal peristalsis in zebrafish larva. (a) Péclet numbers after injection of food into the anterior and posterior intestine. (b) Schematic of food mixing and advection in the zebrafish larval intestine. AI, anterior intestine; MI, middle intestine; PI, posterior intestine; and An, anus. Reproduced with the permission from Kikuchi *et al.*, Am. J. Physiol. **318**, G1013–G1021 (2020). Copyright 2020 APS.

Kikuchi *et al.* (2020) performed real-time *in vivo* imaging of zebrafish larva at 7 dpf for 5 h after feeding and investigated the mechanical roles of anterograde and retrograde intestinal peristalsis in the anterior and posterior intestine. They derived the Péclet number (Fig. 6), which indicates the ratio of advection to diffusion transport. A scaling analysis showed that, after feeding, retrograde peristalsis of the anterior intestine continuously mixes the contents, whereas anterograde peristalsis of the posterior intestine first mixes the contents and then transports them toward the anus. Thus, the role of intestinal peristalsis is drastically changed after feeding.

The zebrafish larval mouth first opens at 74 h post-fertilization (hpf), followed by the anus at 96 hpf, at which point the gut is a continuous tube accessible to the outside environment (Ng *et al.*, 2005). The gut becomes fully functional by 5 dpf, when uptake of lipid and protein begins (Bates *et al.*, 2006). The zebrafish intestinal microbiota composition changes with development (from 4 to 380 days of age) (Stephens *et al.*, 2016). The spatiotemporal transport of the gut chyme and microbiota in the zebrafish intestine was observed by noninvasive microscopy (Bates *et al.*, 2006; Cocchiaro and Rawls, 2013). Jemielita *et al.* (2014) reported that *Aeromonas* in the larval zebrafish intestine exhibits a heterogeneous distribution and aggregates in the middle of the intestinal tract. Wiles *et al.* (2016) investigated the distribution of *Aeromonas* by introducing *Vibrio* into the larval zebrafish intestine. The habitat of *Aeromonas* was modified by competition with *Vibrio*, altering its distribution. The intestinal inflammatory damage in a microbiota-containing larval zebrafish intestine was more extensive than in that of germ-free fish (Qi *et al.*, 2014).

### D. Modeling of the gut flora

Mathematical modeling enables investigation of the distribution and growth of bacteria by clarifying complex phenomena via well-defined numerical experiments. Mathematical models of the gut bacterial flora have been reported (Ishikawa *et al.*, 2011; Cremer *et al.*, 2016; Yang *et al.*, 2018). In this section, we introduce the computational model of Yang *et al.* (2018). They attempted to reproduce the findings of Jemielita *et al.* (2014), in which *Aeromonas* in the larval zebrafish intestine exhibited a heterogeneous distribution and aggregated in the middle of the intestinal tract.

**FIG. 7.**
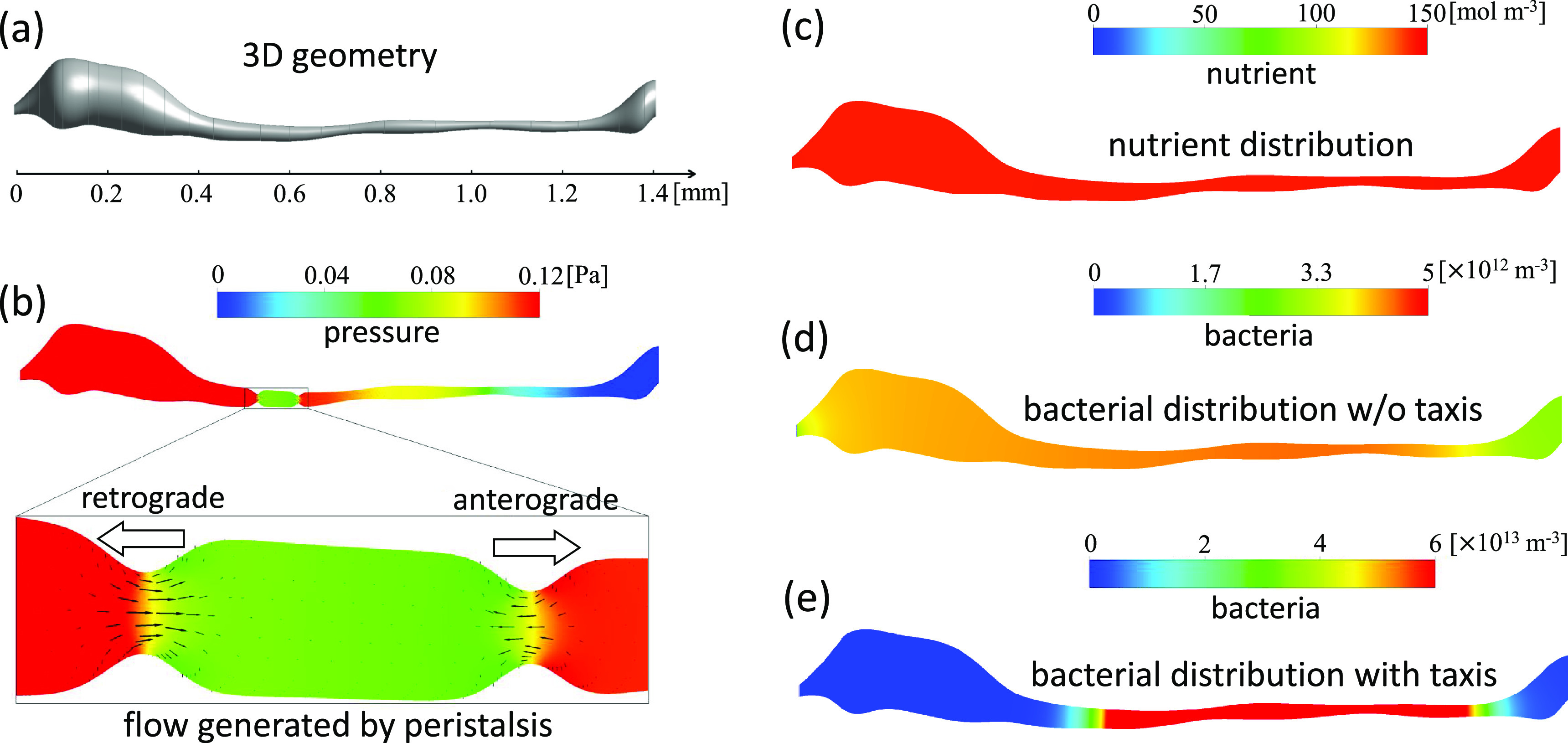
Simulation of the gut flora of a zebrafish larva. (a) Three-dimensional geometry of the intestine. (b) Pressure distribution and velocity vectors. White arrows, retrograde and anterograde peristalsis. (c) Nutrient distribution. (d) Bacterial distribution without taxis. (e) Bacterial distribution with taxis. Reproduced with the permission from Yang *et al.*, J. Theor. Biol. **446**, 101–109 (2018). Copyright 2018 Elsevier.

Yang *et al.* (2018) expressed the distribution and growth of bacteria in the intestine of a zebrafish larva using four conservation laws: (i) conservation of solvent fluid, (ii) conservation of momentum, (iii) conservation of nutrients, and (iv) conservation of bacteria. The results of these equations, however, did not capture the heterogeneous distribution experimentally observed by Jemielita *et al.* (2014) [Figs. 7(a)–7(d)]. In the intestine, bacterial taxis can be triggered by nutrients, pH, temperature, oxygen, antimicrobials, and other factors. Yang *et al.* (2018) mathematically expressed bacterial taxis as a potential field. This enabled reproduction of the heterogeneous distribution [Fig. 7(e)]. The diffusion constants in the governing equations are not equivalent to Brownian diffusivity, because the effects of bacterial swimming need to be considered. To obtain such macroscopic properties, an understanding of the individual and collective motions of cells is needed. This is facilitated by a bottom-up understanding, i.e., from the cellular to the macroscale.

## VI. FUTURE PROSPECTS

In this Review, we introduced recent advances in the biomechanics of bacterial swimming, adhesion to surfaces, collective motion, biofilm formation, and the gut flora. Below, we discuss future prospects in bacterial biomechanics.

Swimming behaviors and chemotaxis of bacteria have been clarified in terms of biomechanics, and their bulk motion in a concentration field can be mathematically described. However, bacterial behaviors in complex environments are unclear. Complex environments have geometric constraints (Ishikawa, 2019), surface chemical properties, background flow fields, concentration fields, and non-Newtonian fluids. Because the physical environment of biofilms and the digestive system is complex, knowledge of bacterial behaviors in complex environments has to be strengthened.

Biomechanics of bacterial adhesion on surfaces has been investigated. However, the interplay among adhesion strategies, including appendages like pili and fimbriae and EPS secretion, has not been fully clarified. The physical environment, such as shear stress, can also strengthen (i.e., catch bonds) or weaken (i.e., slip bonds) bonding strength. To fully understand cell adhesion, experimental data at a cellular resolution have to be accumulated. In addition, our understanding of the population dynamics within a biofilm needs to be improved, because cell division and EPS secretion of adhered bacteria are essential in biofilm formation.

Collective swimming of bacteria has been investigated intensively, and the physical mechanisms of coherent structures emerging within a bacterial bath have been understood. However, bacterial swarming has not been modeled precisely, and several *ad hoc* assumptions have been made. Swarming is a particular biological mode that involves intercellular chemical communication as well as mechanical changes (Beer and Ariel, 2019), which have not been well described mathematically. Transport on an agar gel surface is more complex than that on a solid wall, hampering quantitative investigation of colony-pattern formation. Biomechanics of swarming and colony-pattern formation is an interesting research field to be explored.

Transport phenomena in a biofilm influence cell division and EPS secretion and, consequently, biofilm growth, which in turn influence the surrounding flow field, viscous drag, mass transport, and population dynamics of bacteria. Such crosstalk between bacteria and their environment needs further study. While transport phenomena outside a biofilm can be described mathematically, modeling the population dynamics of bacteria is still challenging. This is in part because (i) we have not obtained sufficient experimental data to establish a mathematical model, (ii) the interactions are complex and contain many variables, and (iii) the parameters in such mathematical models are not well defined or supported experimentally.

Particle-based models can deal with individual bacteria, i.e., cellular resolution. The physical meaning of hydrodynamic, interparticle, and adhesion forces in particle-based models is clear, and the estimation of physical parameters is more straightforward than in continuum models. The drawback of particle-based models is the high computational load required to resolve large tempo-spatial scales. By contrast, macroscopic properties are employed by continuum models, facilitating investigation of large tempo-spatial scales. However, the physical meaning of parameters becomes vague, and *ad hoc* assumptions are often introduced. Combining the advantages of both models will enable development of a model with cellular resolution that can handle large tempo-spatial scales.

Modeling the gut flora is also a challenging task. Approximately 15 000–36 000 bacterial species live in the gut lumen, and an understanding of the interplay between them and the host is needed. A simplified gut flora model would be an important first step. Jemielita *et al.* (2014) inoculated germ-free zebrafish larvae with fluorescently labeled *Aeromonas* species, which is abundant in the zebrafish gut. The presence of a single bacterial species enables interspecies interplay to be ignored. As a second step, Wiles *et al.* (2016) investigated the distribution of *Aeromonas* by introducing another bacterium, *Vibrio*, into the larval zebrafish intestine. This setup enables investigation of the interplay between the two bacterial species. Hence, germ-free zebrafish are expected to provide useful experimental data for modeling of the gut flora.

## Data Availability

The data that support the findings of this study are available from the corresponding author upon reasonable request.
